# Comparison of plant- and mammalian cell-produced human papillomavirus pseudovirions

**DOI:** 10.1016/j.jviromet.2025.115215

**Published:** 2025-06-30

**Authors:** Albertha R. van Zyl, Sarah Lindsay, Georgia Schäfer, Edward P. Rybicki, Inga I. Hitzeroth

**Affiliations:** a Centre for Bioprocess Engineering Research, Department of Chemical Engineering, University of Cape Town, Rondebosch, Cape Town, South Africa; b Biopharming Research Unit, Department of Molecular and Cell Biology, University of Cape Town, Rondebosch, Cape Town, South Africa; c International Centre for Genetic Engineering and Biotechnology (ICGEB), Cape Town, South Africa; d Department of Integrative Biomedical Sciences, University of Cape Town, South Africa; e Institute of Infectious Disease and Molecular Medicine, University of Cape Town, South Africa; f Wellcome Centre for Infectious Diseases Research in Africa, University of Cape Town, South Africa

**Keywords:** HPV, Pseudovirions, PBNA, Luciferase, Plants, Cervicovaginal murine model

## Abstract

High-risk human papillomaviruses (HPVs) are the primary etiological agents of cervical, anal and oropharyngeal cancers. While existing vaccines are effective in preventing infection, their impact in low-and middle-income countries (LMICs) is limited by type coverage, high costs and uptake. To address this gap, there is a critical need for next-generation vaccines that are both regionally tailored and cost-effective, along with efficient and accessible tools for evaluating their efficacy. HPV pseudovirions (PsVs), which encapsidate a reporter plasmid, are widely used in pseudovirion-based neutralisation assays (PBNAs) and *in vivo* murine models to assess vaccine-induced immunity – and have potential for use as DNA vaccine delivery systems. Traditionally, PsVs are produced in mammalian cells, which remain the gold standard due to their high infectivity and structural fidelity. However, recent studies have demonstrated the feasibility of producing PsVs in plants, a platform that offers lower infrastructure and reagent costs, scalability, and biosafety advantages. Although plant-derived PsVs have shown promise in PBNAs, their performance in *in vivo* models had not been evaluated prior to this study. Here, we compared mammalian cell-derived PsVs encapsidating either Gaussia or firefly luciferase reporter plasmids and found that firefly luciferase provided more consistent and robust signals in both *in vitro* and *in vivo* assays. Building on this, we generated PsVs encapsidating the firefly luciferase gene using both mammalian and plant expression systems, and assessed their infectivity. While plant-derived PsVs were capable of infecting HeLa cells and mice in a cervicovaginal challenge model, mammalian-derived PsVs exhibited significantly higher infectivity overall. These findings represent the first demonstration of *in vivo* infectivity of plant-produced HPV PsVs and highlight their potential as a cost-effective alternative for immunogenicity testing and potentially as vaccines. Although further optimization is needed, particularly in capsid assembly and purification, plant-based PsV production holds promise for expanding access to HPV research tools and supporting vaccine development in resource-limited settings.

## 1. Introduction

High-risk human papillomaviruses (HPV) are the etiological agents responsible for the development of cervical cancer, but also other cancers such as oropharyngeal and anal (Bruni et al., 2023; Serrano et al., 2012). Cervical cancer is the fourth most common cancer in women globally, with a high incidence in many low- and middle-income countries (LMICs) due to inefficient implementation of screening and vaccination programmes (Serrano et al., 2018; Singh et al., 2023). Existing HPV vaccines are effective in preventing infection, but their coverage in low- and middle-income countries (LMICs) remains limited, both in terms of vaccine uptake and protection against regionally prevalent HPV types (Guillaume et al., 2024). Expanding vaccine coverage to include additional oncogenic HPV types, such as HPV-35, could enhance cervical cancer prevention strategies in these regions (Denny et al., 2014; Mbulawa et al., 2022; Pinheiro et al., 2020). This necessitates the development of accessible, cost effective methods to evaluate vaccine efficacy, alongside next-generation vaccines tailored for resource-limited settings (Sanders et al., 2023). The World Health Organisation (WHO) has emphasized the importance of these strategies as part of the global initiative to eliminate cervical cancer, particularly in LMICs where the burden is highest (Bruni et al., 2021; Zou and Zhang, 2023).

Papillomaviruses are species-specific and cannot easily be cultured *in vitro* (Campo, 2002); therefore, no *in vivo* or *in vitro* challenge models using native viruses exist for evaluating efficacy of HPV vaccines. As an alternative, pseudovirion-based neutralisation assays (PBNAs) and transgenic murine models are commonly used to evaluate vaccine-induced immune responses (Sanders et al., 2023). HPV pseudovirions (PsVs) are virus-like particles (VLPs) consisting of the L1 and L2 capsid proteins that can encapsidate up to 8 kb of non-papillomaviral DNA, such as a reporter plasmid or a pseudogenome (Buck et al., 2004; Buck et al., 2005). While PsVs and VLPs have similar capsid structures composed of L1 or both L1 and L2, VLPs are empty capsids lacking genetic material and form the basis of current prophylactic vaccines (Huang et al., 2020). In contrast, PsVs encapsidate DNA, enabling their application in gene delivery and neutralising assays. PsVs are crucial in PBNAs used to evaluate immunogenicity of vaccines, and in particular to test for neutralising antibodies (Buck et al., 2005; Pastrana et al., 2005). Previous studies have also investigated the use of papillomavirus PsVs as vectors for plasmid delivery across various cell types, in gene therapy applications, and for the mucosal delivery of DNA vaccines (Bayer et al., 2018; Brendle et al., 2021; Cerqueira et al., 2017; Çuburu et al., 2012; Gilson et al., 2020; Graham et al., 2010; Kines et al., 2015; Ma et al., 2011).

Murine cervicovaginal challenge models utilising PsVs have been developed and have been successfully used to investigate HPV infection mechanisms and to evaluate protective immune responses elicited to vaccine antigens (Handisurya et al., 2012; Johnson et al., 2009; Longet et al., 2011; Roberts et al., 2007; Sanders et al., 2023). To produce high titres of PsVs traditionally, HEK293TT cell lines are co-transfected with L1 and L2-encoding plasmid(s) along with a reporter gene plasmid (Buck et al., 2005). The use of HEK293TT cells for the production of PsVs presents some limitations, including the requirement for specialized and costly equipment and reagents, that must be operated within biosafety level 2 (BSL-2) laboratory settings. Additionally, since HPV capsids can encapsidate any heterologous DNA smaller than 8 kb, there is a potential biosafety risk of inadvertently packaging oncogenic sequences, such as the SV40 large T antigen gene, which has been shown to cause cellular transformation (Ma et al., 2011).

Transient expression of proteins in *Nicotiana benthamiana* (*N. benthamiana*) plants provides a viable, less time-consuming and more scalable alternative to mammalian expression systems, with an essentially zero risk of contamination with mammalian pathogens and endotoxins (Fischer et al., 2004; Marsian and Lomonossoff, 2016; Rybicki, 2010). HPV proteins, and in particular VLPs of several HPV types composed of the L1 capsid protein, have been expressed in plants, and vaccination of animals with these VLPs induced humoral immune responses (Chabeda et al., 2019; Kohl et al., 2006; Matić et al., 2012; Naupu et al., 2020; Pineo et al., 2013). Moreover, it has been demonstrated that HPV and bovine papillomavirus (BPV) PsVs can be produced in plants through co-infiltration of plant expression vectors encoding the L1 and L2 capsid proteins, along with a replicating vector encoding a reporter gene. These plant-derived PsVs were able to encapsidate reporter gene plasmid DNA, successfully infected HEK293TT cells, and were effectively utilised in PBNAs. In these studies, enhanced green fluorescent protein (eGFP) and secreted alkaline phosphatase (SEAP) reporter genes were encapsidated in the plant-derived PsVs. However, direct comparisons between plant- and mammalian cell-produced PsVs were not conducted (Adams et al., 2023; Lamprecht et al., 2016; Pietersen et al., 2020). Therefore, in this study, we aimed to produce PsVs encapsidating a single reporter gene, luciferase, in both plant and mammalian expression systems, and to evaluate their performance in a head-to-head *in vivo* murine cervicovaginal infection model. In addition, we first determined which luciferase gives a clearer, more distinct signal by comparing mammalian cell-derived PsVs encapsidating either Gaussia or firefly luciferase reporter plasmids in *in vitro* and *in vivo* models.

## 2. Materials and methods

### 2.1. Maintenance of HEK293TT and HeLa cell lines

The human embryonic kidney cell line, HEK293TT (ATCC CRL-3467) and the human cervical adenocarcinoma cell line HeLa (ATCC CCL-2) were used to produce and to test the mammalian cell-made PsVs, respectively (Buck et al., 2005). Both cell lines were grown in Dulbecco’s Modified Eagle Medium (DMEM, Life Technologies) supplemented with 10 % heat-inactivated foetal calf serum (FCS, Gibco), 100 U/mL penicillin and 100 μg/mL streptomycin (Pen/Strep) in Corning^®^ T75 cell culture flasks (Merck). Cells were grown and maintained at 37°C with 5 % CO_2_ and 95 % humidity and passaged at 70 % confluency.

### 2.2. HEK293TT-cell PsV production and purification

HEK293TT cells were seeded in Corning^®^ T175 flasks (Merck) containing DMEM supplemented with FCS and Pen/Strep and incubated as described above until they reached approximately 70 % confluency. Cells were co-transfected with codon optimised pXULL-HPV-16L1/L2 (kindly provided by John Schiller, National Institutes of Health, Bethesda, MD, USA) and either of the following reporter gene plasmids, pGL3-control (Promega) and pCMV-GLuc (New England Biolabs) harbouring firefly (FLuc) and Gaussia luciferase (GLuc) genes, respectively.

HEK293TT cells seeded the previous day in 10-cm dishes were transfected using the calcium phosphate transfection method following published procedures (Buck et al., 2005; Jordan et al., 1998). Briefly, per 10-cm cell culture dish, 5 μg pXULL plasmid and 12 μg reporter gene plasmid (either pGL3-control or pCMV-Gluc) was mixed with 2.5 M CaCl₂ in a total volume of 200 μL TE buffer. Subsequently, 2X HEPES-buffered saline (HBS) was added dropwise to form DNA-calcium phosphate precipitates. The mixture was incubated for 30 min at room temperature and then applied to HEK293TT cells cultured at 50–80 % confluency and incubated for 48 h before cell lysis for PsV extraction.

Cells were lysed using 10 % Brij58 in PBS (supplemented with 9.5 mM MgCl_2_, benzonase and Exonuclease V to remove all unpackaged DNA), and incubated for 24 h at 37°C for virus maturation. After addition of 0.17 volumes 5 M NaCl, cell lysates were freeze/thawed three times and thereafter clarified by centrifugation at 8000 × *g* for 10 min at 4°C. The PsV-containing supernatants were collected and layered onto 2-step discontinuous CsCl gradients consisting of 0.27 g/mL CsCl and 0.39 g/mL CsCl dissolved in HSB buffer (25 mM HEPES, pH 7.5, 0.5 M NaCl, 0.02 % Brij58, 1 mM MgCl_2_, 100 μM EDTA, 0.5 % ethanol). Gradients were ultracentrifuged for 16 h at 20 000 × *g* at 4°C in a Beckman Coulter L8–55M ultracentrifuge using a SW40Ti swinging-bucket rotor to allow for separation of PsVs, empty capsids and protein aggregates (Smith et al., 2007). Opaque bands containing PsVs at the heavy/light interface and approximately 400 μL of the more dense CsCl phase were collected after centrifugation. To facilitate concentration of the PsVs and removal of CsCl through buffer exchange, the collected fractions were loaded onto Amicon Ultra-4 filter devices (100 kDa MWCO, Merck) and centrifuged at 3000 × *g* for 10 min. HSB buffer was added to the filter devices and the samples centrifuged again to enable complete removal of CsCl. The PsV-containing samples were concentrated to a final volume of approximately 100 μL. PsVs were stored in low-binding siliconized tubes at −80°C. Bradford assays (Bio-Rad) were carried out to determine the PsV concentration relative to a bovine serum albumin (BSA, Bio-Rad) standard curve.

### 2.3. Plant-based PsV production and purification

For plant-based production of HPV-16 PsVs, pTRAkc-rbcs1-CTP (Maclean et al., 2007) containing either the HPV-16 L1 or L2 human codon optimised capsid genes (pTRA-CTP-hL1 and pTRA-CTP-hL2, respectively), and a replicating expression vector, pRIC3.0 (Regnard et al., 2010), containing the firefly luciferase (FLuc) reporter gene were co-infiltrated into *N. benthamiana* plants.

Recombinant *A. tumefaciens* GV3101::pMP90RK colonies harbouring the above mentioned constructs were inoculated into 10 mL LBB medium (2.5 g/L tryptone, 12.5 g/L yeast extract, 5 g/L NaCl, 1.95 g/L MES, pH 5.6) supplemented with 50 μg/mL kanamycin and 50 μg/mL carbenicillin and incubated with shaking for 18 h at 27°C. The cultures were sequentially scaled up to 50 mL and finally 500 mL using the conditions described above. The final 500 mL cultures were supplemented with 200 μM acetosyringone (Sigma) in addition to the antibiotics to allow for the induction of the *vir* genes prior to infiltration. The cultures were diluted in resuspension solution (0.975 g/L MES, 2.03 g/L MgCl_2_.6H_2_O, pH 5.6) and infiltrated into 6–8 week old *N. benthamiana* plants by vacuum infiltration as previously described (Lamprecht et al., 2016).

Biomass was harvested at 4 days post infiltration (dpi) after which PsVs were purified on Optiprep^™^ (Sigma-Aldrich) gradients as previously described (Lamprecht et al., 2016). To facilitate removal of Optiprep^™^ and concentration of the PsVs, particle-containing fractions were pooled and loaded onto Amicon Ultra-4 filter devices (100 kDa MWCO, Merck) and centrifuged at 3000 × *g* for 10 min. PBS (2 mL) was added to the filter devices followed by another round of centrifugation to enable complete removal of Optiprep^™^ from the PsVs. PsVs were concentrated to a final volume of 2 mL and stored in low-binding siliconized tubes at −80°C.

### 2.4. Confirmation of the presence of L1 and L2 in purified PsVs

Samples of both the mammalian cell- and plant-produced PsVs were denatured by heating to 95°C in the presence of 1X sample application buffer (5X SAB: 100 mM TrisCl pH 7.5, 2 % SDS, 2 mM EDTA, 25 % glycerol, 4.3 % β-mercaptoethanol) for 10 min. Denatured proteins were resolved on 10 % SDS-PAGE gels at 180 V using a Mini-PROTEAN Tetra Cell (Bio-Rad). The Colour Protein Standard, Broad Range (10–250 kDa, New England Biolabs) was used as molecular weight marker. For detection of L1 and L2, gels were stained using the ProteoSilver^™^ Stain kit (Sigma-Aldrich) according to the manufacturer’s instructions. The gels were fixed overnight in 100 mL fixing solution (50 % ethanol, 10 % acetic acid) to ensure better resolution and lower background detection.

### 2.5. Transmission electron microscopy

Purified PsVs were visualized using transmission electron microscopy (TEM). Copper carbon-coated grids were made hydrophilic by glow discharging at 25 mA for 30 s using a Model 900 SmartSet Cold Stage Controller (Electron Microscopy Sciences). The carbon side of the grids were floated on a 20 μL drop of sample for 5 mins. The grids were washed 3x with filter sterilized dH_2_O and negatively stained for 1 min with 2 % (w/v) uranyl acetate. Grids were viewed at 27 000 – 50 000x magnification using a FEI Tecnai 20 electron microscope fitted with a 200 kV LaB6 emitter.

### 2.6. Rolling circle amplification of encapsidated DNA

Purified PsV samples (20 μL) were treated with 1 μL of a 1/10 dilution of proteinase K (Merck) to digest the capsid proteins, resulting in the release of any encapsidated DNA. The reaction was carried out at 55°C for 3 h after which the proteinase K was inactivated by heating the reactions to 95°C for 10 min. Rolling circle amplification (RCA) of the purified PsVs was performed using the illustra^™^ Templiphi 100 Amplification Kit (GE Healthcare) according to the manufacturer’s instructions. Briefly, 1 μL of the proteinase K-treated samples were added to 5 μL sample buffer, the samples were denatured for 3 min at 95°C and cooled to 4°C. A master mix containing 5 μL reaction buffer and 0.2 μL enzyme mix per reaction was prepared, 5 μL of the master mix was added to each sample. RCA was carried out for 18 h at 30°C, after which the samples were heated to 65°C for 10 min to stop the reaction. RCA reactions were subsequently linearised with the *Hind*III (Thermo Fisher Scientific) restriction enzyme for 1 h at 37°C. The digested samples were analysed on 1 % agarose gels stained with ethidium bromide; the O’GeneRuler 1 kb Plus DNA Ladder (Thermo Fisher Scientific) was used as molecular weight marker.

### 2.7. In vitro infectivity assays

The infectivity of PsVs was tested in HeLa cells through *in vitro* assays. HeLa cells, a human cervical adenocarcinoma cell line, are commonly used to evaluate the infectivity of PsVs. HeLa cells were seeded in 48 well plates (Thermo Fisher Scientific) at a density of 1 × 10^5^ cells per well and incubated overnight. PsVs were neutralised by incubation with rabbit-raised Gardasil^®^ antiserum that was produced in-house (Naupu et al., 2020) for 30 min at room temperature; a control that was not treated with antibody was also evaluated. The neutralised and untreated PsVs were added to the HeLa cells at a final viral density of approximately 0.5 μg/cell and incubated at 37°C for 48 h. The media was collected for further analysis, and the cells were mechanically lysed with a cell scraper in the presence of 50 μL Luciferase Cell Culture Lysis Reagent (Promega). The media and lysates (30 μL) were transferred to white 96 well plates and luciferase activity was measured using the Pierce^™^ Gaussia Luciferase Glow Assay Kit (Thermo Fisher Scientific) or the Luciferase Assay System (Promega), respectively, according to the manufacturer’s instructions. Luminescence was determined using a GloMax^®^ Explorer Multimode Microplate Reader (Promega). Raw luciferase data were normalised against the uninfected cells-only wells. The experiments were performed in triplicate.

### 2.8. In vivo infectivity of PsVs in mice

Approval for this study was obtained from the Animal Research Ethics Committee at the Faculty of Health Sciences at the University of Cape Town (UCT, AEC 021–023). Six-weeks-old female Balb/C mice were randomly divided into three groups consisting of two experimental groups with five mice each and a control group with three mice. Mice were treated with 2 mg/20 g Depo-Provera (Pfizer) suspended in 200 μL PBS by subcutaneous injection to ensure equilibration of hormonal levels and to prolong dioestrus which facilitates infection of sexually transmitted diseases (Kaushic et al., 2003). Four days later, mice were anaesthetized via intraperitoneal injection of 125 μL ketamine (1.5 mg/20 g) and xylazine (0.2 mg/20 g), and cervicovaginal epithelial cells were chemically disrupted by intravaginal delivery of 25 μL of 4 % Nonoxynol-9 (N-9, Abcam) in a formulation of 3 % carboxymethylcellulose (CMC) (Roberts et al., 2007). Mice were allowed to recover for 6 h after which they were anaesthetized as described above and intravaginally inoculated with 5 μg of mammalian cell-produced PsVs encapsidating either FLuc or GLuc, or 5 μg of plant-produced PsVs encapsidating FLuc in a total volume of 20 μL in the presence of 3 % CMC.

The genital tracts of mice infected with PsVs encapsidating GLuc were lavaged twice with 50 μL sterile PBS to collect secreted Gaussia luciferase at 24 h, 48 h, and 72 h post infection. Cells and debris were removed from the washings by centrifugation at 3000 × *g* for 5 min, and the supernatants were analysed for secreted Gaussia luciferase expression as a measure for infection using the Pierce^™^ Gaussia Luciferase Glow Assay Kit (Thermo Fisher Scientific). Luminescence was determined using a GloMax^®^ Explorer Multimode Microplate Reader (Promega). To measure infection with PsVs encapsidaing FLuc (mammalian cell- and plant-produced), mice were anaesthetised by inhalation of isoflurane and intravaginally injected with 50 μL luciferase reagent (Luciferase Assay System, Promega). Light emission was measured using an IVIS^®^ Spectrum *in vivo* imaging system (Perkin Elmer). The readings obtained from the IVIS^®^ Spectrum scanner are reported as average radiance (p/sec/cm^2^/sr) which normalises for the area over which the luminescence is measured.

### 2.9. Statistical analysis

When comparing two groups, multiple unpaired parametric t-tests were employed. For more than two groups, two-way ANOVA with Dunnett’s or Tukey’s multiple comparison tests, respectively, were performed. Significant differences are indicated with a * when p < 0.05, ** when p < 0.01, *** when p < 0.001, and **** when p < 0.0001. All calculations were performed with Graph Pad Prism (Version 9).

## 3. Results

### 3.1. In vitro comparison of PsVs made in mammalian cells encapsidating different luciferase reporter genes

**Fig. 1.**
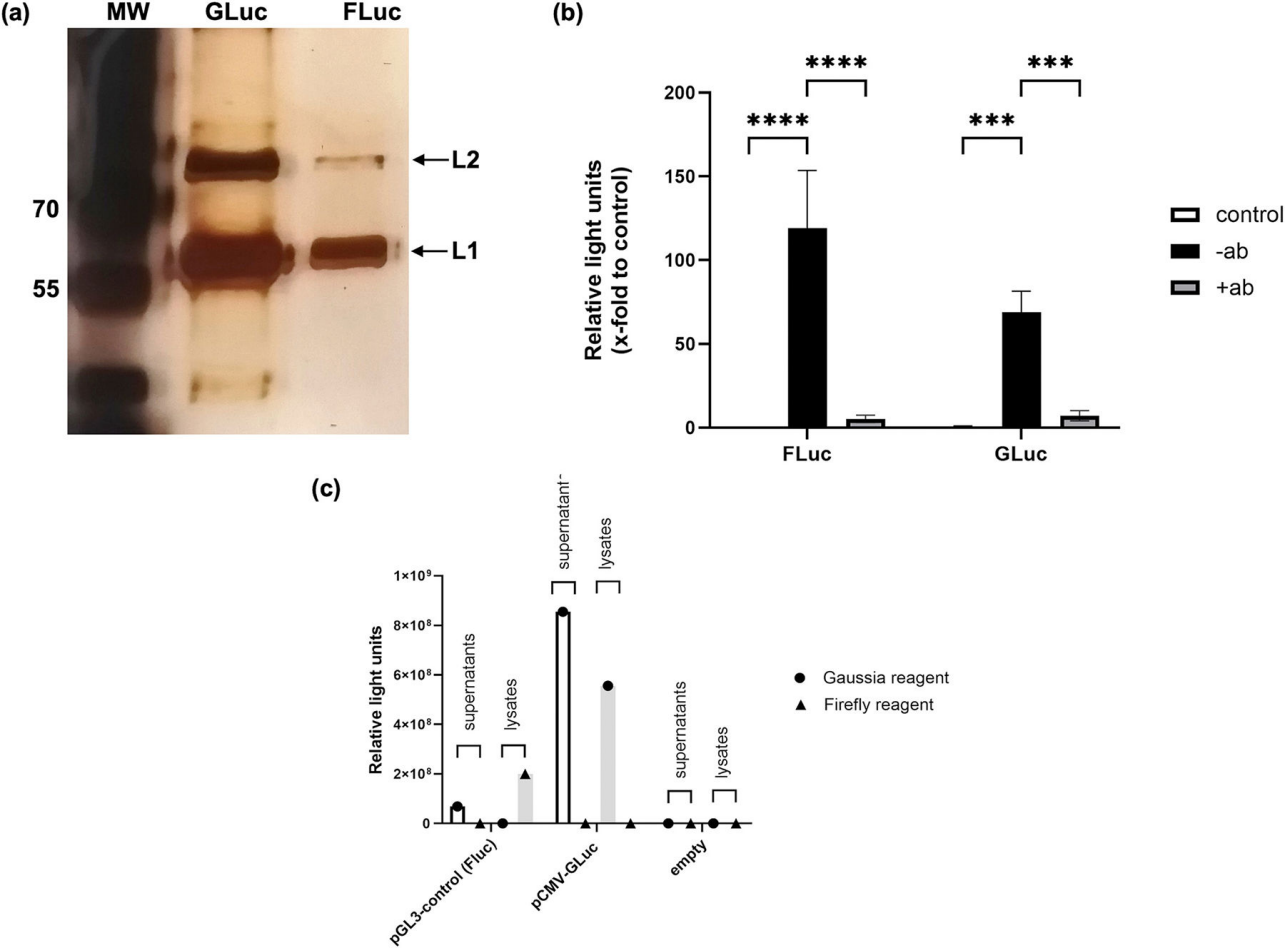
Confirmation of L1 and L2 expression and in vitro infectivity analysis in HeLa cells. a) Silver-stained SDS-PAGE gel of purified PsVs, equal volumes of purified PsVs were loaded in each lane. L1 and L2 bands are indicated by the arrows with L1 and L2 detected at approximately 56 kDa (bottom arrow) and 84 kDa (top arrow), respectively. MW, molecular weight marker. b) In vitro infectivity (no antibody: -ab, black bars) and neutralisation assays (with antibody: +ab, grey bars) of PsVs encapsidating GLuc and FLuc were carried out in HeLa cells. Luciferase assays were conducted 48 h post infection on cell supernatants (GLuc) or cell lysates (FLuc), respectively. Luciferase readings are shown as x-fold increase compared to uninfected controls. A two-way ANOVA with Tukey’s multiple comparison test was used to test statistical significance between groups. c) HeLa cells were transfected with PsVs to compare cross-reactivity and sensitivity of the luciferase reagents for detection of GLuc and FLuc in supernatants and cell lysates. Cellular supernatants or cell lysates derived from transfections of either the pGL3-control plasmid (expressing FLuc) or the pCMV-GLuc plasmid (expressing GLuc) were tested for FLuc and GLuc activities using the respective reagents as indicated. Untransfected cells served as negative control.

We first compared mammalian cell-derived PsVs encapsidating either Gaussia or firefly luciferase reporter plasmids to determine which luciferase provided more consistent signals *in vitro*. Silver-stained SDS-PAGE gels of mammalian cell-produced PsVs encapsidating Gaussia luciferase (GLuc) or firefly luciferase (FLuc) revealed distinct L1 and L2 bands at approximately 56 kDa and 84 kDa, respectively (Fig. 1a). PsV infectivity was assessed 48 h post infection by measuring secreted GLuc in HeLa cell supernatants and FLuc in cell lysates. Luciferase assays confirmed expression of both GLuc and FLuc in HeLa cells, thereby serving as a proxy for successful PsV infection (Fig. 1b). PsVs encapsidating GLuc and FLuc were successfully neutralised by rabbit antiserum raised against Gardasil^®^, as demonstrated by the reduced reporter gene expression (Fig. 1b), indicating specific surface display of neutralising epitopes. To confirm the localisation of FLuc and GLuc and assess potential cross-reactivity of luciferase reagents, HeLa cells were transfected with the respective reporter plasmids. The firefly luciferase reagent specifically detected FLuc, without cross-reactivity to GLuc. In contrast, the Gaussia reagent produced background signals in supernatants from FLuc-expressing cells, indicating some non-specific activity. FLuc was exclusively detected in cell lysates, confirming its intracellular retention, whereas GLuc was present in both supernatants and cell lysates, consistent with its secretion and partial intracellular retention (Fig. 1c).

### 3.2. In vivo comparison of luciferase reporter genes

**Fig. 2.**
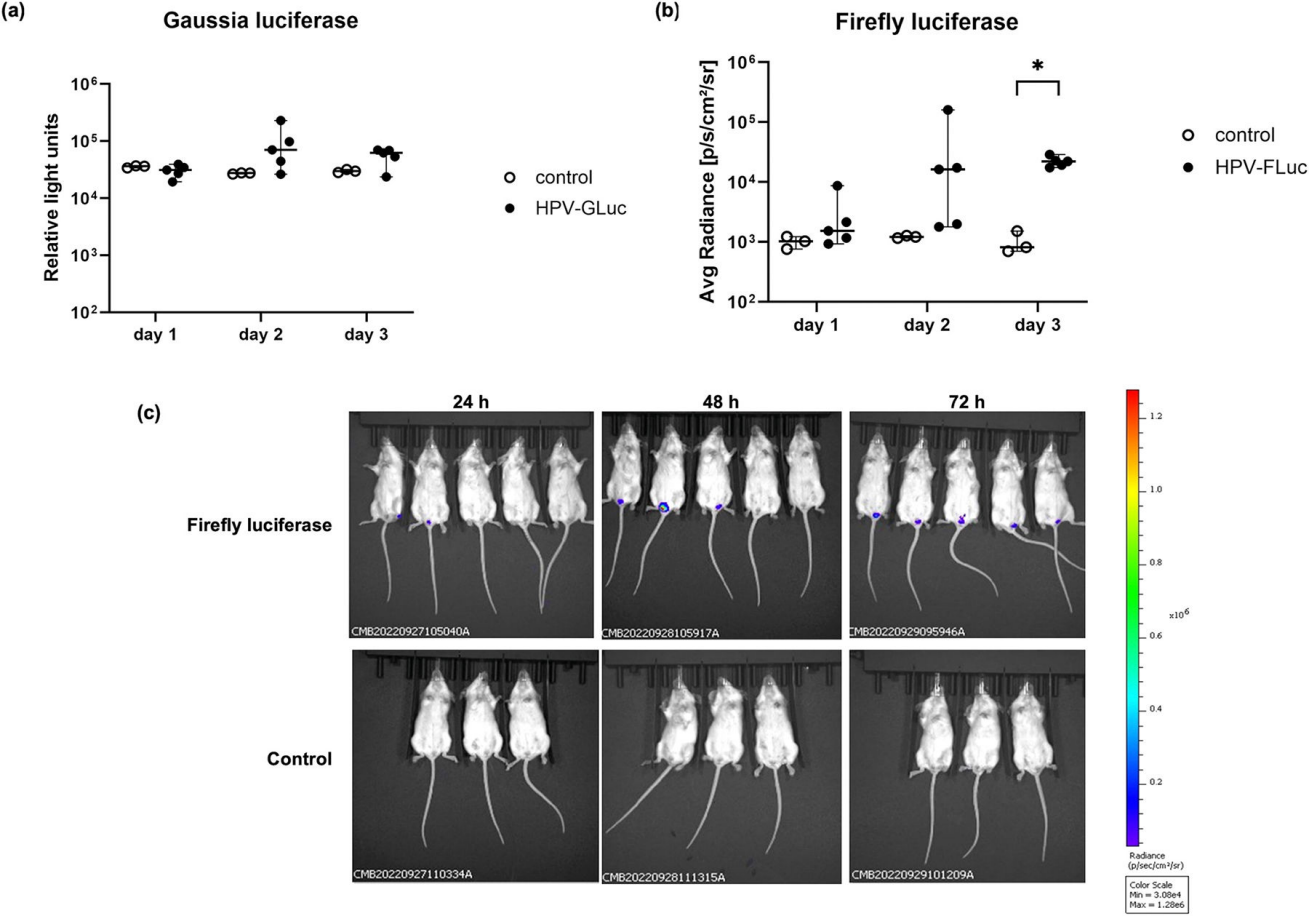
In vivo infectivity of PsVs encapsidating GLuc and FLuc. Mice were infected with 5 μg of PsVs. a) Gaussia luciferase detection in vaginal lavages of mice infected with PsVs encapsidating GLuc (in relative light units) showing infection of individual mice at 24 h – 72 h post infection. The horizontal black bars represent the average for each group, with error bars showing the standard deviation. An unpaired parametric *t*-test showed no significant difference between the experimental and control groups. b) Firefly luciferase detection of mice infected with PsVs encapsidating FLuc (expressed as radiance/total flux readings) obtained using the IVIS^®^ Spectrum scanner of individual mice at 24 h – 72 h post infection. Analysis to test for significance between control and experimental groups were carried out using an unpaired parametric *t*-test. c) Images of PsVs-FLuc infected mice (top panel) compared to negative control mice (bottom panel). Mice were placed in the same position for imaging at the different time intervals.

Similarly, we compared the mammalian cell-produced PsVs encapsidating either Gaussia or firefly luciferase genes in mice to determine which one was more suited to use. Mice infected with PsVs expressing secreted GLuc were vaginally lavaged every 24 h over a 72 h. As shown in Fig. 2a, no GLuc expression was detected at 24 h post infection. By 48 h post infection, four of five mice showed successful infection, although luminescence readings showed large variance. At 72 h, GLuc was consistently detected in four of the five infected mice, while uninfected controls showed no signal throughout the study (Fig. 2a).

Mice infected with PsVs expressing the intracellularly retained FLuc were imaged every 24 h for 72 h to monitor peak firefly luciferase expression in the cervicovaginal area (Fig. 2b and c). At 24 h post infection, only one of five mice showed a bioluminescent signal; this increased to three out of five mice showing successful infection by 48 h post infection. As with GLuc, substantial variance was detected in the luminescence readings obtained 48 h post infection. By 72 h post infection, all five mice exhibited luciferase expression with reduced variance in readings (Fig. 2b and c). No luminescence was detected in negative controls throughout the 72 h period (Fig. 2b and c).

While we demonstrated that PsVs encapsidating both GLuc and FLuc reporter plasmids were successfully produced in HEK293TT cells and were able to infect HeLa cells, our results indicated that FLuc detection was more specific. FLuc activity was confined to cell lysates when the appropriate substrate was used, whereas GLuc assays produced background signal in FLuc-expressing cells and was shown to be present in both the supernatant and cell lysates analysed (Fig. 1c). In addition FLuc allowed *in vivo* imaging of mice instead of using invasive procedures such as vaginal lavage that were required to analyse for GLuc expression. Therefore, to compare infectivity of plant- and mammalian cell-produced PsVs, FLuc was selected as reporter gene.

### 3.3. Confirmation of PsV assembly and reporter plasmid encapsidation

**Fig. 3.**
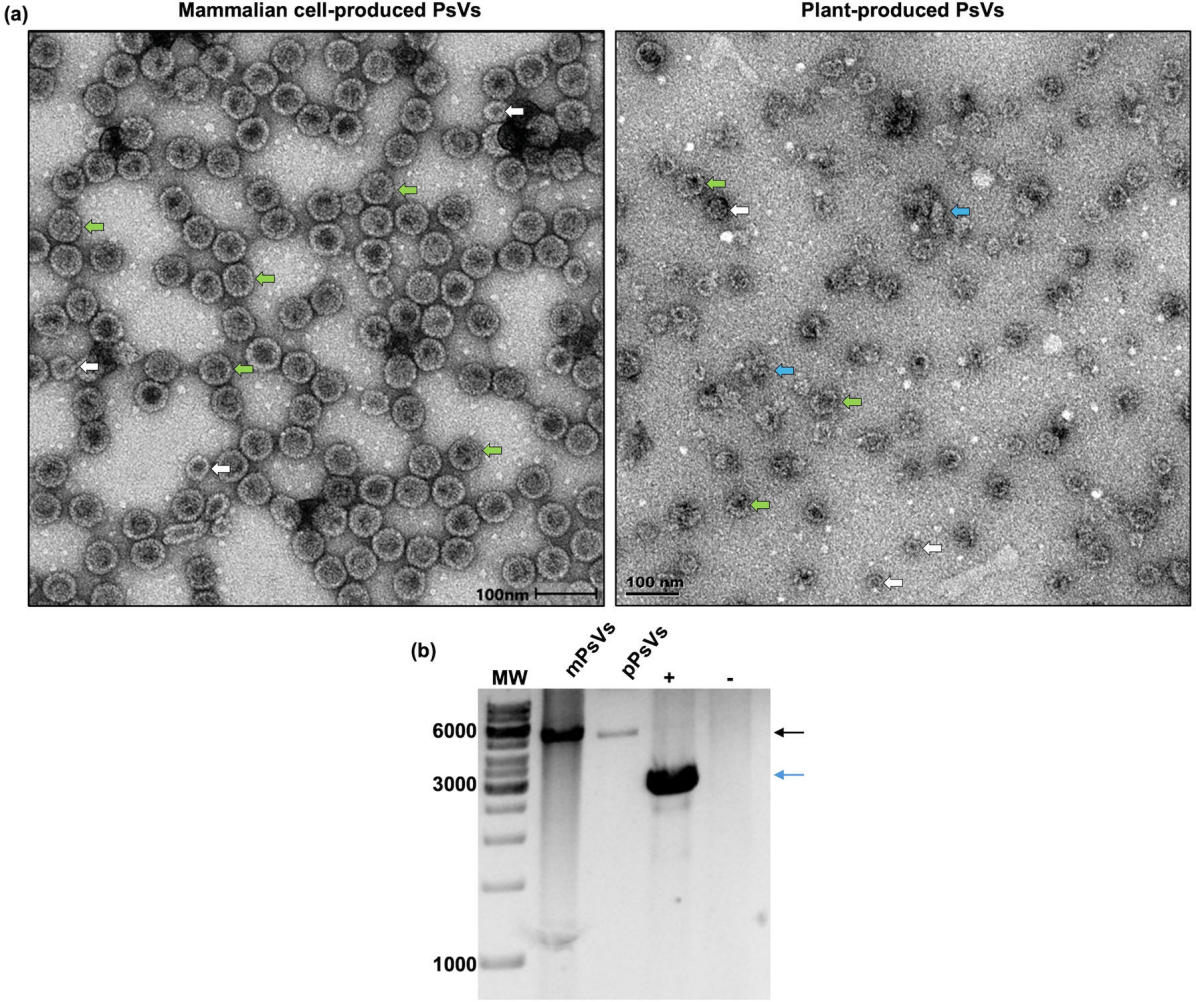
Confirmation of particle formation and encapsidation. a) TEM analysis of purified and concentrated mammalian and plant-produced PsVs. Green arrows: PsVs 50 – 60 nm in diameter, white arrows: PsVs 20 – 40 nm in diameter, blue arrows: capsomeres and protein aggregates. Scale bar: 100 nm, magnification: 27 000 – 53 000x. b) RCA and restriction enzyme digest confirm encapsidation of reporter plasmid DNA in PsVs. Linearised reporter plasmid: black arrow, linearised pUC19 positive control plasmid (+): blue arrow, no template negative control: (−), MW: O’GeneRuler 1 kb Plus DNA Ladder (Thermo Fisher Scientific).

To compare mammalian cell-produced and plant-derived PsVs encapsidating FLuc on a structural level, TEM analysis and RCA was performed. TEM analysis (Fig. 3a) of the mammalian cell-produced PsVs showed the presence of fully formed PsVs ranging from 40 – 60 nm in diameter. In comparison, electron micrographs of plant-produced PsVs showed the presence of smaller particles and partially formed capsids, ranging in size from 20 – 50 nm. These results are indicative of incomplete or aberrant assembly. The plant-produced particles observed were irregularly shaped and TEM analysis further showed the presence of protein aggregates and capsomeres.

To evaluate successful encapsidation of the FLuc reporter plasmid, purified mammalian cell- and plant-produced PsVs were subjected to RCA following disassembly. Hind*III* digestion of the RCA product yielded a fragment of approximately 5100 bp in both cases, indicating successful encapsidation of plasmid DNA. A band of greater intensity was observed for mammalian cell-produced PsVs, indicating higher DNA encapsidation compared to the plant-produced PsVs (Fig. 3b). The pUC19 vector served as positive RCA control and digestion with *Hind*III resulted in the expected band observed at approximately 2600 bp (Fig. 3b), while no bands were detected in the negative control.

### 3.4. Comparison of mammalian cell- versus plant-produced PsV infectivity

**Fig. 4.**
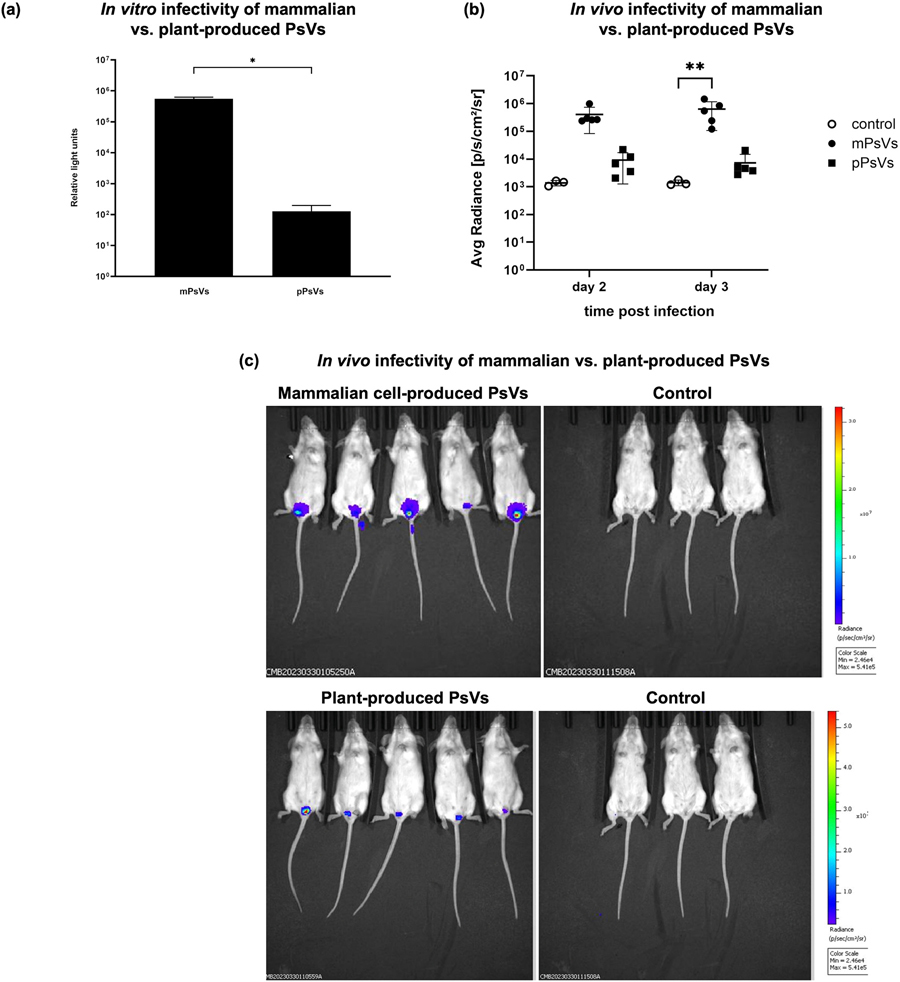
Infectivity of mammalian cell- and plant-produced PsVs encapsidating FLuc. Mice were infected with 5 μg of PsVs. a) Infectivity of PsVs in HeLa cells 48 h post infection. Log of the relative light units are plotted; the error bars indicate the standard deviation. Analysis to test for significance between control and experimental groups were carried out using an unpaired parametric *t*-test. b) Bioluminescence imaging data of the average radiance/total flux readings (reported in relative light units) obtained using the IVIS^®^ Spectrum scanner of individual mice at 48 h and 72 h post infection. A two-way ANOVA with Dunnett’s multiple comparison test was used to test statistical significance between groups. c) Bioluminescent imaging of mice infected with PsVs compared to negative control mice 72 h post infection. The same control mice were used for both mPsVs and pPsVs; however, the radiance/colour scale was adjusted for detection of luminescence in mice infected with plant-produced PsVs.

To compare infectivity of the mammalian cell- versus plant-produced PsVs encapsidating Fluc, HeLa cells were infected with equal amounts of the virus preparations, based on total protein content. FLuc assays showed higher infectivity (as measured by reporter gene expression/activity) with mammalian cell-produced PsVs (Fig. 4a). *In vivo*, mice infected cervicovaginally with the PsV preparations showed notably higher and more consistent fluorescent signals at 48 h and 72 h post infection in the mammalian PsV group compared to the plant-produced PsV group (Fig. 4b). At 72 h post-infection, all five mice infected with mammalian cell-produced PsVs showed fluorescent signals, while only four of the five mice in the plant PsV group showed successful infection. On average, luminescence from plant-produced PsVs was approximately 100-fold lower than that from mammalian cell-produced PsVs (Fig. 4c).

## 4. Discussion

HPV PsVs encapsidating reporter genes encoding reporters such as luciferase, GFP or SEAP are widely used to study the mechanisms of HPV infection *in vitro* and *in vivo* (Buck et al., 2005; Ma et al., 2011). *In vivo* challenge models using these PsVs have facilitated drug and vaccine testing, enabling real-time imaging of infection and immune protection in mice and rabbits (Campo, 2002; Longet et al., 2011; Ma et al., 2011; Sanders et al., 2023).

Selecting an appropriate reporter gene depends on several factors, such as stability of the reporter gene, signal intensity, and whether the protein is secreted or intracellular (Neefjes et al., 2021). Firefly luciferase, a commonly used intracellular reporter gene, is ideal for locational imaging (Gould and Subramani, 1988; Hakkila et al., 2002). In contrast, Gaussia luciferase is secreted, allowing for non-destructive, real-time monitoring and high throughput analysis, and is particularly useful when specialised imaging equipment is not available (Hiramatsu et al., 2005).

HPV PsVs are typically produced in HEK293TT cells and require co-transfection of L1, L2 and reporter plasmids. While L2 is not required for capsid formation, it is critical for DNA encapsidation and viral entry into host cells (Buck et al., 2008; Holmgren et al., 2005; Okun et al., 2001). In this study, transduction and reporter protein expression of mammalian cell-produced PsVs encapsidating either FLuc or GLuc were compared. PsVs were produced using the same L1 and L2 expression vectors, but the firefly and Gaussia luciferase reporter genes were harboured on different plasmids. As encapsidation depends on DNA size rather than sequence, differences in reporter plasmids were not expected to affect packaging efficiency (Adams et al., 2023; Buck et al., 2004; Cerqueira and Schiller, 2016; Xu et al., 2006; Zhao et al., 1998). Both PsVs encapsidating GLuc and FLuc successfully infected HeLa cells, and neutralisation assays confirmed that neutralising epitopes were effectively displayed on the surface of the PsVs (Fig. 1b). As expected, FLuc was detected only in cell lysates, while GLuc was present in both the supernatants and cell lysates, consistent with its secretory nature. Notably, FLuc detection was more specific, as GLuc assay reagents showed some cross-reactivity with FLuc-expressing samples (Fig. 1c).

Following *in vitro* validation, transduction of PsVs encapsidating firefly and Gaussia luciferase were evaluated in a mouse model. While direct comparison of signal intensity between FLuc and GLuc is not feasible, FLuc yielded more consistent luciferase signals (Fig. 2a and b). GLuc readings showed greater variability, likely due to variations in vaginal washings collected and the influence of complex bodily fluids on assay performance (Hiramatsu et al., 2005; Neefjes et al., 2021). Additionally, GLuc is not completely secreted and remains partly intracellular, contributing to signal inconsistency. FLuc enabled more consistent bioluminescent imaging of mice, with less heterogeneity in data collected (Fig. 2b and c). This can be attributed to FLuc being fully retained within cells (Gould and Subramani, 1988). Heterogeneity is a known limitation in these *in vivo* models. Sanders et al. (2023) investigated whether the addition of detergent to prevent PsV aggregation or CMC to increase viscosity and limit drainage of PsVs from the vaginal vault could improve the consistency of bioluminescent signal obtained. However, signal variability persisted, likely due to PsV aggregation, host-specific differences between the individual mice and natural resistance to vaginal inoculation (Sanders et al., 2023). Given its higher specificity, lower variability and compatibility with non-invasive imaging, FLuc was selected for subsequent experiments. However, in resource-limited settings without access to an IVIS instrument, measuring GLuc from vaginal lavages remains a viable alternative, albeit with higher variability that may require large experimental group sizes to ensure statistical robustness.

Plant-based expression systems are promising platforms for producing HPV PsVs, offering potential advantages in scalability and biosafety. Previous studies from our group demonstrated that plant-produced HPV PsVs were able to infect mammalian cells and could also be used in PBNAs (Adams et al., 2023; Lamprecht et al., 2016). In this study, we directly compared plant- and mammalian-derived PsVs encapsidating the FLuc-expressing plasmid. While mammalian cell-produced PsVs assembled into particles resembling native HPV virions and HPV PsVs produced in mammalian cells (Anisimová et al., 1990; Buck et al., 2005; Kim et al., 2016), plant-derived PsVs displayed larger heterogeneity, including the presence of aggregates and capsomeres. These findings were consistent with earlier reports (Adams et al., 2023; Lamprecht et al., 2016). Despite these structural differences, both types of PsVs encapsidated, or were associated with, the reporter plasmid, as confirmed by RCA (Fig. 3b).

While the presence of encapsidated DNA suggests successful production of functional PsV formation, transduction efficiency remains a more reliable indicator of functionality. In HeLa cells, mammalian cell-produced PsVs yielded significantly higher firefly luciferase activity than plant-produced PsVs (Fig. 4a). *In vivo*, mammalian cell-produced PsVs demonstrated significantly higher infectivity than plant-derived PsVs, with luciferase expression levels approximately 100-fold greater at 48 h and 72 h post infection when compared to plant-made PsVs (Fig. 4b and c). Although equal amounts of L1 were used for infection, the amount of encapsidated reporter DNA was not determined. The performance of mammalian-produced PsVs may be attributed to their more uniform morphology, which likely enhances binding to the extracellular matrix (ECM) and facilitates efficient L2 cleavage and subsequent DNA delivery (Culp et al., 2006; Day et al., 2007; Horvath et al., 2010). In contrast, the pleomorphic nature of the plant-produced PsVs may hinder these early infection steps. Additionally, previous studies have shown that nuclear factors enhance production of infectious PsVs during *in vitro* assembly (Cerqueira et al., 2017; Cerqueira and Schiller, 2016), suggesting that differences in nuclear components between plant and mammalian cells may contribute to inefficient assembly and reduced infectivity of plant-derived PsVs. This disparity probably reflects the same structural and functional limitations observed *in vitro*, particularly the reduced ability of plant-derived PsVs to bind and penetrate the basement membrane (Ma et al., 2011).

Differences in the production pipelines likely contribute to these disparities. In plants, successful PsV formation requires co-infiltration of three recombinant *Agrobacterium* strains, each carrying a separate construct. In contrast, HEK293TT cells co-express L1 and L2 from a single plasmid, with the reporter plasmid provided separately, enabling more efficient co-delivery and expression. Furthermore, mammalian cell-produced PsVs undergo a maturation step after cell lysis to enhance assembly, capsid stability and DNA encapsidation (Buck et al., 2005). Plant-produced PsVs were not matured, as extended incubation of crude plant extracts led to L1 degradation despite the use of protease inhibitors (results not shown). While mammalian cell-produced PsVs were purified using CsCl gradients, plant-derived PsVs were processed with Optiprep^™^ due to the large extract volumes. The use of different density media may have affected virion stability, but CsCl purification was not feasible for plant-derived material. These methodological differences, along with intrinsic differences in cellular machinery, probably affect particle assembly and stability in plant systems. Optimisation of expression, maturation and purification methods will be essential to improve the quality and consistency of plant-produced PsVs.

This study is the first demonstration that plant-produced HPV PsVs can successfully infect mice. While mammalian-derived PsVs consistently outperformed plant-derived PsVs in terms of morphology, infectivity, and reporter expression, this work identifies key areas for optimisation in plant-based production. The potential advantages of using this approach are substantial. Plant systems offer a scalable, cost-effective and biosafe alternative to mammalian cell culture, with lower infrastructure and reagent costs, making them particularly attractive for use in low-resource settings. Future work should focus on improving co-expression strategies (e.g. expression of L1 and L2 from a single construct), developing maturation methods compatible with plant extracts and refining purification methods to enhance particle quality. PsVs from other papillomavirus types have already been made in plants (Pietersen et al., 2020; Adams et al., 2023), which further validates the utility of the approach. By identifying both the limitations and the promise of plant-based PsV production, this study lays the groundwork for an accessible and adaptable system for HPV research.

## Data Availability

No data was used for the research described in the article.
